# Review of Digital Economy Research in China: A Framework Analysis Based on Bibliometrics

**DOI:** 10.1155/2022/2427034

**Published:** 2022-08-08

**Authors:** Yanmin Xu, Yitao Tao, Chunjiong Zhang, Mingxing Xie, Wengang Li, Jianjiang Tai

**Affiliations:** ^1^Shenzhen Institute of Administration, Shenzhen 518034, China; ^2^China Special Economic Zone Research Center, Shenzhen University, Shenzhen 518061, China; ^3^Department of Computer Science, Tongji University, Shanghai 201804, China; ^4^School of Humanities, Shanghai University of Finance and Economics, Shanghai 200433, China

## Abstract

This article combs the present situation and future development trend of digital economy research in China by means of literature measurement. On the one hand, China's digital economy presents a framework system of industrial digitization, digital industrialization, digital governance, and data value. On the other hand, digital economics has gradually become a dominant science and is constantly challenging the traditional economic research framework. Data are no longer the traditional data, and the data-driven economic and social innovation development model is the future trend. Data privacy, data protection, data ethics, data assets, and so on will become the focus of academic research.

## 1. Introduction

The global economy is undergoing digital transformation. With the frequent interaction of digital trade around the world, governments, enterprises, and other subjects are facing digital upgrading. Data have increasingly become a new factor of production, which makes the operation mechanism of the world economy show new characteristics. The socialized application of digital technology has brought the rise of digital society and shaped the form of digital economy characterized by data. The development of digital economy drives the high-quality development of social economy. We should focus on the people, promote the sharing of the dividends of digital economy development among the people, and contribute to the people's sense of gain and happiness, so as to build a community with a shared future in digital space and realize a new global shared digital economy.

From the perspective of the development of the theory of the digital economy, the concept of the digital economy has been deepening from the information economy to the concept of the digital economy. In the 1940s, there was a breakthrough in the field of microelectronics. In the 1980s and 1990s, digital technology and information network technologies converged to drive the development of digital economy, and traditional information technology models and business models changed. This has driven the rapid development of digital technologies such as cloud computing and big data. The concept of the digital economy was first proposed by the American scholar Don Tapscott in 1996, followed by the publication of a study on the emerging digital economy by the United States Department of Commerce. Recent years have seen explosive growth in publications related to China's digital economy, such as “Introduction to the Digital Economy: Theory, Practice, and Strategy,” published in 2022 by the China Institute of Information and Communication Research and “Theory and Governance of the Digital Economy” (2021) by Dong Yang and Xinyu Xu. Based on this, this article focuses on the related literature on digital economy research in China through CNKI database. Based on the analysis of the whole article, author, and journal, this article will provide some references and inspiration for digital economy research, and further support the discipline construction, social application, and future trend of digital economy.

## 2. Research Design and Overall Analysis

### 2.1. Data Sources and Research Methods

This article takes CNKI database as the data source, selects unlimited time, and selects Peking University core journals, CSSCI journals, and CSCD journals according to the theme = “digital economy.” As of April 29, 2022, 4060 documents have been retrieved. After literature processing, a total of 3643 literature were selected as sample data, excluding the lack of author, title, keyword, and meeting notice.

This article uses CNKI database, VOSviewer, SPSS, and other software to visually analyze the research status of digital economy in China. Specifically, one is to make statistics on the annual production of digital economy papers. The second is to count the number of articles published by authors and journals. The third is to draw the systematic clustering map of high-frequency keywords and analyze the situation of related topics. Through the above research, this article combs and analyzes the development trend and theme context of digital economy.

### 2.2. Time Distribution Analysis

**Figure 1:**
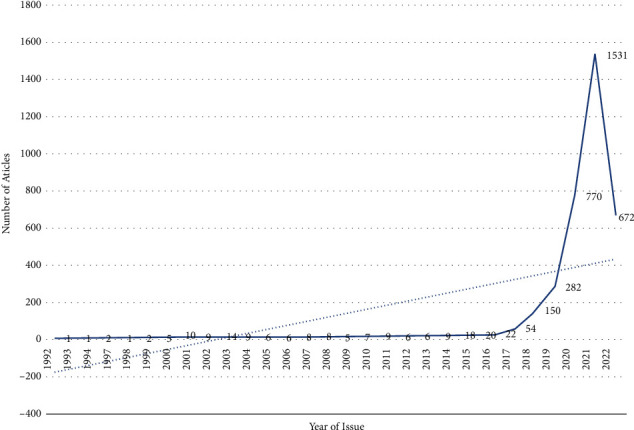
Time distribution of literature.

Through the time distribution of digital economy research literature in China, we can intuitively judge the development of academic research on digital economy. As shown in Figure 1, the historical trend of document issuance is roughly divided into three stages. In the first stage (1992–1998), the literature volume basically fluctuated between 1 and 2 articles, and even no articles on relevant topics were published from 1995 to 1996. In the second stage (1999–2016), the number of literature fluctuated between 5 and 22, showing a stable trend. The third stage (2017–2020) showed steady growth and began to show exponential growth in 2018, of which the number of documents issued in 2021 reached a historical peak. It can be seen that the overall trend of China's digital economy research is extremely hot and shows a blowout situation.

### 2.3. Author Statistical Analysis

**Figure 2:**
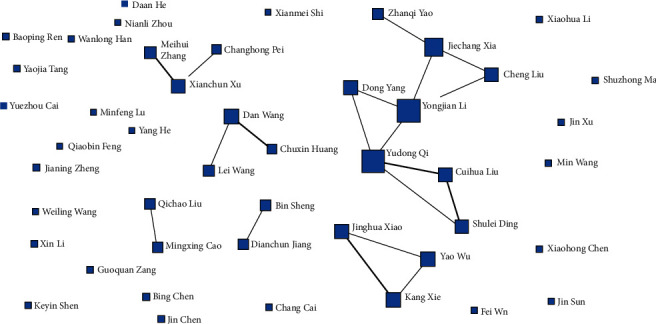
Co-occurrence map of authors.

Using UCINET software to draw the co-occurrence chart of authors (Figure 2), a total of 49 authors have published more than (including) 6 articles (including cooperation; Table 1). Among them, the top five authors are Yudong Qi (25 articles), Bing Chen (23 articles), Dong Yang (20 articles), Baoping Ren (16 articles), and Jiechang Xia (16 articles). Academic cooperation includes teams with Yudong Qi as the core (including Cuihua Liu, Shulei Ding, Yongjian Li, Dong Yang, Cheng Liu, Jiechang Xia, and Zhanqi Yao), cooperation teams of Xianchun Xu, Changhong Pei, and Meihui Zhang, and cooperation among Lei Wang, Chunxin Huang, and Dan Wang; cooperation between Qichao Liu and Mingxiao Cao. There is a cooperation between Dianchun Jiang and bin Sheng, and other authors mostly work alone, which reflects the current situation of domestic digital economy research aggregation research to a certain extent.

### 2.4. Journal and Keyword Analysis

As shown in Table 2 the distribution of journals, 29 Chinese digital economy research journals have published more than 20 articles (including 20 articles), among which the top 5 journals are *Journal of Commercial Economics* (100 articles), *International Taxation in China* (98 articles), *Taxation Research* (98 articles), *People's Tribune* (74 articles), and *China Finance* (58 articles). *Journal of Commercial Economics* is a journal focusing on the field of commercial circulation; *International Taxation in China* and *Taxation Research* focus on the frontier of economic tax theory and practice, and pay close attention to the research progress of digital economy and digital tax; *People's Tribune* is a comprehensive publication, and *China Finance* is a financial journal, focusing on the experience of financial reform and discussing economic and financial theoretical issues.

A total of 3643 literature were extracted from the keyword field and measured by co-occurrence [1] (hereinafter referred to as COOC) software carries out word frequency statistics and extracts 7288 keywords, including 67 keywords with frequency ≥20 (Table 3). Use COOC software to generate 67 high-frequency keywords × 67 co-occurrence matrix (Table 4), due to layout constraints, some high-frequency keywords will be displayed. In order to facilitate hierarchical cluster analysis, the keyword co-occurrence matrix is transformed into dissimilarity matrix by COOC software (Table 5).

### 2.5. Hierarchical Cluster Analysis

**Figure 3:**
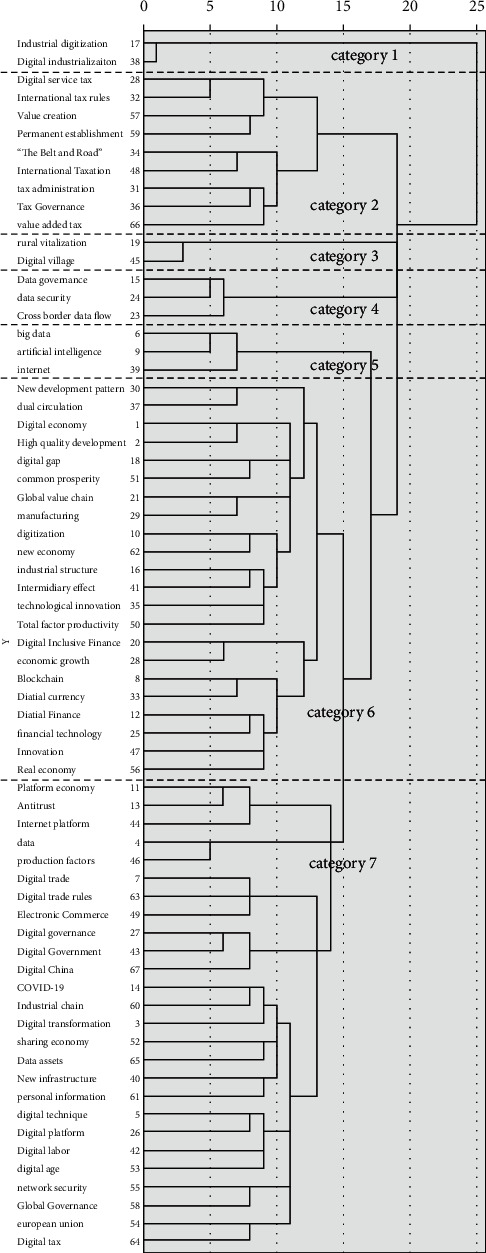
Visual diagram of high-frequency keyword clustering.

For the content theme of China's digital economy research, the dissimilarity matrix of high-frequency keywords in the sample literature (Table 5) is introduced into SPSS software for systematic clustering (Figure 3), which can be roughly divided into seven thematic clusters: first, around the two categories of digital industrialization and industrial digitization; second, focus on national macro policies, such as the Belt and Road and international tax rules; third, rural revitalization and digital rural construction; fourth, focus on data governance and cross-border data flow; fifth, research on embedding digital economy around big data, artificial intelligence, Internet of things, and other technologies; sixth, research on the integrated development of digital economy and traditional real economy, including keywords such as technological innovation, real economy, manufacturing, common prosperity, high-quality development, and so on; and seventh, research on the theory and practice of digital economy, including keywords, platform economy, digital platform, digital governance, digital government, data, digital China, and digital age.

## 3. Subject Content Analysis

Through the literature keyword cluster analysis diagram of China's digital economy, the corresponding content framework system of China's digital economy research is obtained. As shown in Figure 3, according to the keyword clustering, the digital economy research is summarized into seven categories, which are introduced below.

### 3.1. Research on Industrial Digitization and Digital Industrialization

Cluster 1 includes keywords industrial digitization and digital industrialization. Digital industrialization is the basis of the development of digital economy and the leading force of the reform of digital economy innovation system. Industrial digitization is an important part of digital economy, which brings efficiency improvement by integrating the application of digital technology into traditional industries. Du [2] believes that the key breakthrough of digital industrialization should be promoted in an unbalanced way, the full development of industrial digitization should be promoted in an integrated way, and the coordinated development of digital industrialization and industrial digitization should be promoted in a matching way. Data as a factor of production are different from traditional factors of production. It has the characteristics of non-exclusivity and replicability. China needs to improve the participation and distribution mechanism of data factors, promote the transformation of economy, efficiency, and power, and then promote the digital upgrading of industry [3].

The development of digital economy relies on the digital transformation of traditional industrial structure and the construction of digital technology-related industrial systems, so as to achieve mutual synergy between the two and drive high-quality economic and social development. In particular, the convergent innovation and development of new technologies such as artificial intelligence and 5g is an important symbol of the digital economy, which has expanded various digital application scenarios, reconstructed people's production relations and lifestyle, and finally, provided a technical framework for the arrival of the digital era. For example, Internet companies such as Tencent, Huawei, Alibaba, and TikTok have become leading enterprises in the digital economy industry, driving the digital transformation of China's economy and contributing to the construction of Digital China.

### 3.2. Research on Macro Policy and Taxation

Cluster 2 focuses on macro policy environment research, including keywords digital service tax, international tax rules, value creation, “the belt and road,” international tax, tax administration, tax governance, and value-added tax. The international and domestic dual cycle development pattern is the content of the national 14th five-year plan, which promotes the development of China's digital economy from the two-way development of supply and demand. To a certain extent, take double circulation as the starting point, actively promote the development level of digital technology, promote the digital synergy of industrial chain, and then improve the governance system of digital economy, build corresponding institutional system, and promote the balanced development of digital economy [4].

The digital wave has become the main melody of the global economy. Especially in the post-epidemic era, governments all over the world are using digital intelligent technology to participate in the epidemic control and the allocation of people's living materials. The construction of digital innovation value system is related to international competition and national strategic development. Improving digital technology innovation capability, promoting the digital transformation of traditional industries, and strengthening platform system governance have become important directions for the construction of China's digital economy ecosystem.

Data transactions create value for the digital economy and challenge the current tax collection system. China lacks corresponding tax system for data transactions, and digital tax has become a barrier to the service trade of data media to a certain extent. For data transactions with strong value creation ability but scattered distribution, through the comparison of digital economy tax systems of the United States and the European Union, provide ideas for the construction of tax system for localized data transaction [5]. The principles of digital tax legislation should be based on the four principles of difference, economy, regulation, and taxability, reveal the necessity and feasibility of digital tax legislation, and clarify the internal relationship between the value and norms, objectives, and means of legislation. Through the consideration of fairness and efficiency, order and freedom, and security and development, it will help the above three pairs of value guidance, provide value choice for the synergy between the legislative principles of digital tax, and then promote the generation of digital tax system [6].

### 3.3. Research on Digital Village Construction

Cluster 3 focuses on the research of digital village construction. The keywords include rural vitalization and digital village. The key to comprehensively promoting rural revitalization lies in the revitalization of rural industry, which provides a development opportunity for the construction of digital countryside. Digital technology infrastructure is the hardware foundation for the development of rural industries. Through the construction of digital logistics infrastructure, we can reconstruct the spatial pattern of urban and rural areas, accelerate the flow of urban and rural factors, enable the development of rural industries with digital technology, promote the integration of primary, secondary, and tertiary industries in rural areas, promote the diversified and comprehensive development of rural industries through the cultivation of new industries and new business forms, and attract and cultivate new rural digital farmers. Let farmers become the development subject and beneficiary subject of rural industrial prosperity, and form an endogenous driving force to promote the all-around revitalization of rural areas [7].

China's digital village construction is an inevitable measure of rural revitalization in the new era. The “siphon benefit” of digital economy enables the construction of digital countryside, which lays a foundation for promoting the construction of digital countryside in four aspects: digital industrialization, industrial digitization, digital governance, and data value. We will explore four ways to promote the construction of “digital infrastructure and practice” in rural areas, and build a “digital infrastructure and practice” model, so as to comprehensively promote the construction of rural infrastructure and implement digital governance. We will explore four ways to promote the “digital infrastructure and practice” and promote the construction of rural infrastructure and implement the “digital governance” [8].

### 3.4. Research on Data Protection and Governance

Cluster 4 focuses on digital protection and governance. Keywords include data governance, data security, and cross-border data flow. Data elements drive the economy to produce a multiplier effect, promote enterprises' efficient decision-making, improve efficiency, drive innovation, and realize the orderly development of public governance. Data elements need corresponding mechanisms to release their potential value. Data governance system ensures that data elements drive economic development. Among them, data governance policy system includes data privacy protection, data property rights, data competition, and regulatory policies [9]. The protection mode and supervision mechanism of personal privacy data are related to the orderly development of digital economy. It is not the best strategy only from the market mechanism or government supervision. It is necessary to construct a privacy protection system with the linkage of government supervision and market mechanism; According to the traditional national experience, the single emphasis on the concept of private contract or the concept of personality right protection is not the best solution, but the combination of personality right protection and private contract. The government supervision system is a system that balances multiple objectives and the relationship between multiple stakeholders. Privacy protection needs to improve the ability of individual privacy protection and prevention, and strengthen the subject responsibility of enterprises and the contract responsibility between multiple subjects [10].

The development of digital economy drives economic and social transformation. In order to ensure that the legal system is consistent with economic and social development, the reform of the legal system should avoid rigidity, adhere to the principles of inclusiveness and prudence, pay attention to social co-governance, establish a regular evaluation system, and promote the reform of the government supervision system [11]. From the perspective of national legislation, the legislative approach to government data opening is the practical need to ensure the efficient circulation of data elements and release digital dividends. The legislative concept should ensure the balance between the efficiency and security of data openness. The legislative framework should take openness as the main line, ensure the scope and standards of government data openness, construct the confidentiality review and security management system of data openness, standardize the management and construction of data openness platform, and specify the corresponding responsibilities, so as to continuously optimize the business environment of digital economy and realize digital China [12]. Data risk management and control is one of the risks faced by the reform in the digital age. The traditional governance paradigm is not enough to deal with the risk problems in the new era. Develop from the perspective of national security, ensure the top-level design of data legal system design for data security legislation, and deal with risk management and control through data security protection system, so as to ensure national data security risks. Establish the identification and identification system of important data, stipulate the security protection obligations of important data processors, incorporate the security review of important data into the network security review, and establish the exit control system of important data [13]. With regard to data ownership and usufruct, which is related to data property rights and the construction of data market mechanism, the control, development, licensing, and transfer of data should be carried out under the principles of nondiscrimination, fairness, and rationality [14]. In view of the definition of data element property rights and the establishment of income distribution mechanism, we need to analyze the production process of data elements, that is, the ownership of data property rights should belong to data producers. With the help of market mechanism, the market subjects of original data should be provided with appropriate compensation. Data elements should participate in distribution, while taking into account fairness and efficiency, so as to promote the sharing of development achievements by the whole people [15].

### 3.5. Research on Digital Economy Supported by Digital Intelligent Technology Embedding

Cluster 5 focuses on digital intelligence technology, and the keywords are big data, artificial intelligence, and the Internet. Disruptive digital intelligence technology has changed the traditional social and economic structure system. Starting from technological progress and enterprise operation and development, there is an internal relationship between enterprise digital transformation and enterprise innovation. Digitization is the external motivation to promote enterprise innovation, and enterprise innovation is the internal demand to realize digital transformation. To change the management thinking mode and re-recognize the relationship between people and technology, we need to clarify the development objectives and create a good innovation atmosphere, improve the business ability of enterprises, and provide corresponding countermeasures and suggestions for the innovation of enterprise development model [16]. The collaborative integration process among digitization, industrialization, and ecology promotes the economic development of socialism with Chinese characteristics [17], digital technology innovation system and infrastructure level affect the change of division of labor status in the global value chain. While improving digital productivity, speeding up the construction of digital governance system, and protecting the rights and interests of ordinary workers, avoid digital monopoly and digital discrimination, promote social equity, and improve social welfare [18].

The analysis framework of organizational resilience looks at the development of new formats of digital economy, ensures that the retail industry has resilience in the uncertain process, presents different segmentation capabilities in different stages, and explores the development status and capacity-building of new formats of retail through live broadcasting and social e-commerce [19]. Xiao et al. published a series of articles on the innovation logic of digital economy, including data-driven product innovation [20], adaptive innovation of big data cooperative assets [21], adaptive innovation of data-driven organizational structure [22], adaptive innovation of data-driven technology contract [23], digital transformation and adaptive innovation of production mode [24], that is to investigate the digital economy through digital products, digital technology, digital production structure, and production mode.

### 3.6. Research on the Integration of Digital Economy into the Development of Traditional Real Economy

Cluster 6 focuses on the research on the integration of digital economy into the development of traditional real economy. The keywords include new development pattern, dual circulation, digital economy, high-quality development, digital gap, common prosperity, global value chain, manufacturing, digitization, new economy, industrial structure, intermediate effect, technical innovation, total factor productivity, digital inclusive finance economic growth, financial technology, innovation, real economy, etc. The digital transformation of the economy under the epidemic provides new impetus for development, deepens reform, and promotes the construction of a community of shared destiny system. In the process of domestic and international double cycles, we use data elements as the starting point to help the high-quality development of the digital economy, meet the needs of the people, and lay the foundation for the new pattern of China's economic development [25]. However, the high-quality development of China's digital economy still faces many restrictive factors, mainly including weak research and development of primary innovation of digital economy, low openness of digital economy to the outside world, weak voice in global digital economy governance, and so on. In order to promote the high-quality development of China's digital economy, we must promote the balanced development of digitization among and within industries, strengthen the opening-up of the field of digital economy to the outside world and the inside world, give each government and market its place, and strive to optimize the business environment for the high-quality development of digital economy [26].

Look at the digital economy from the macro-, meso-, and micro-dimensions. From the macro analysis, the application of big data expands the boundary of resource allocation in the planned economy and contributes to the coordination between the government and the market. From this point of view, the innovative mechanism of digital economy has changed the traditional market structure and power structure, forcing buyers and sellers to trade under the mode of complete competition. From the micro perspective, the digital economy integrates economies of scale and breaks the traditional economic profit model [27]. The shape of the global value chain presents an uncertain picture, which requires rethinking the unbalanced situation of the world's economic development pattern, so as to build a global resource allocation and benefit distribution balance based on the flow of factors. It is urgent to solve the contradiction between the free flow of factors and the digital divide, and correct the income mismatch of the data value chain [28].

In the digital age, enterprise boundary breakthrough has become a development trend, and its evolution approach and theoretical logic are rooted in the fact that information and knowledge have become key core production factors in the digital age, which is very important for enterprise core competence and expansion power. Specifically, the core competence of enterprises has changed from the control of tangible resources to the sharing of intangible resources. Data driven has become the key logic for the development of innovative business, that is, enterprises cultivate the ability of data acquisition and mining, so as to contribute to their digital transformation and innovative development [29]. The regulatory mechanism needs to be matched with the development of the digital economy. We can no longer use the traditional regulatory mechanism to meet the ever-changing development requirements, establish a perfect regulatory legal system, improve the dual center collaborative governance subject construction system of platform + government, maintain the principle of inclusiveness and prudence, promote sharing and openness, encourage innovation, maintain the law of market operation, and ensure the dynamic and effective regulatory mechanism and policies [30]. Product adaptive innovation has the characteristics of high-frequency interaction, real-time feedback, and user leadership. It forms a multi-party interaction mechanism among products, users, and enterprises. Through the mining of user needs, it can quickly match user needs, so as to realize adaptive innovation [31].

Digital economy promotes the refinement of the global value chain division system, breaks through the original time and space constraints, and promotes the evolution of the global value chain division system [32]. Starting from the global economy and trade, the digital trade brought by the digital economy has impacted the traditional trade attributes and brought corresponding governance challenges. The existing multilateral trade agreements lack effective regulations on digital trade barriers and digital risks. The existing framework classification of trade in goods and trade in services requires open rules and measures for the trade of digital products compatible with the attributes of trade in services and goods. In particular, the restrictions on data circulation caused by data circulation barriers, the prohibition of facility localization, and the mandatory disclosure of source code are the regulations to deal with the data circulation of digital trade. To a certain extent, it causes harm to national security and consumer privacy, giving the government policy space to control data risks [33].

### 3.7. Research on Theoretical System and Specific Practice of Digital Economy

Cluster 7 focuses on the research of digital economy theory and specific practice. The keywords include platform economy, antivirus, Internet platform, data, production factors, digital trade, digital trade rules, electronic commerce, digital governance, digital government, digital China, Covid-19, industrial chain, digital transformation, sharing economy, digital platform, digital labor, etc. In view of the construction of discipline framework systems such as discipline orientation, research methods, and research contents of digital economics, this article views the transformation and development of discipline paradigm of digital economics from the dimension of technological innovation, attaches importance to the cultivation of digital economy talents, and then constructs the academic community of digital economy research [34]. Digital economics not only contributes to the digital transformation of social production mode, but also helps to reshape the basic logic of economic management theory. Combined with the connotation characteristics of digital economy, the non-competitiveness of data elements, and the unity of production and consumption, this article explores the social changes brought by digital technology and the changes of narrative paradigm of economic theory, so as to contribute to the research of digital economics.

The research on the connotation and discipline development framework of digital economy theory is based on the paradigm of technical economy and the theory of complex economics. The development of digital technology drives the innovation of economic research methodologies, such as machine learning and federal learning, which are applied to economic research, promotes the updating of traditional measurement methods, and deeply excavates the laws of economic activities and economic periodicity with the help of big data, thus contributing to the development of digital economics. Digital economy is a complex of various economic activities with intelligent technology group as the driving force and data as production factors, including driving force, new form structure, value creation activities, etc. The uncertainty of technological evolution, the unknownness of social development, the lag of policies and systems, and the prominence of digital sovereignty limit the development of the technological economy paradigm of digital economy. The development of digital economy will inevitably bring the iterative and phased integration of the economic development paradigm. Starting from the principles of coordination and balance, promote the breakthrough of basic theory, improve the top-level design, and accelerate the development of new economy from the construction of technical facilities and institutional adaptation [35].

Technological revolution has shaped new forms of economy, such as intelligent economy, experience economy, sharing economy, space economy, etc., which requires not only the construction of new economic forms in theory but also the direction of transformation in practice. The new economic form is not a negation of the original economic form, but an innovative development. It is the transformation, optimization, and promotion of the old economic system by the new technology group [36]. With the application of digital technology in the economic system and the transformation and upgrading of the enabling currency system, digital currency came into being. International private digital currency causes inflation or deflation and subverts the traditional monetary payment system, which will weaken the status of sovereign currency. China should speed up the landing of legal digital currency, promote the internationalization of RMB, and explore the development model of China's digital currency [37]. In the development of digital economy, we should pay equal attention to making up for weaknesses and casting longboards, consumer and industrial ends, efficiency and inclusiveness, market mechanism and institutional advantages, and create an environment for the high-quality development of digital economy on the basis of constantly optimizing institutional mechanisms and policy systems [38].

## 4. Conclusion

The digital economy is the frontier of current academic research and the subject of multidisciplinary integration research. This article studies the literature on digital economy in CNKI database; draws the knowledge map with the help of VOSviewer, SPSS, UCINET, and other software; analyzes the author co-occurrence, keywords, and other maps; and combs the current situation of digital economy research in China, theme context, and other topics. Through the structural analysis of the categories of China's digital economy research, this study reflects the current research status in stages to some extent, but there are some errors in the keyword classification effect, such as the intersection of macro policies and specific practical problems to some extent. For the specific practical process, we need to summarize and predict the future development. The author summarizes the future research direction in combination with high-frequency hot words, The details are as follows.

Firstly, the research system of digital economy has become a reality according to the framework of digital industrialization, industrial digitization, digital governance, and data value. To a certain extent, this reflects the current situation of digital economy research. In the future, the transformative development of digital economy innovation system will drive the qualitative change of social-spatial structure and corresponding functions. It will analyze the mechanism changes of digital technology, digital trade, digital finance, digital government, and digital security from the dimensions of economy, culture, politics, and technology, so as to provide strategic support and corresponding guidance for global economic governance [39].

Secondly, the digital economy brings about the overall digital transformation of social and economic space, and moves toward daily, specific, and subtle. In the process of digital integration, building a people-centered digital social space and improving people's digital literacy will become an important topic for future research. For example, rural digital transformation needs to reshape the countryside in an integrated and all-around way, and drive the transformation of rural development mode with the help of digital intelligent technology. This requires not only the digital driving force of rural economic and industrial structure but also the digital reshaping of the overall rural operation system. Clarify the purpose and mode of digital transformation. While empowering urban development, the final value appeal should reflect the value appeal of the people, and finally, truly realize the sharing of development achievements among the people.

Thirdly, from the perspective of discipline development, the discipline of digital economy is generating its own unique narrative paradigm, becoming a discipline, and constantly challenging the traditional economic research framework. The development framework of digital economy discipline includes the connotation, essential characteristics, evolutionary development mode, dynamic mechanism, driving mechanism, policy system, experience reference, development dilemma, and optimization path of digital economy. To a certain extent, the development of digital economy discipline will lead the development direction of economic theory in the future.

Finally, digital sovereignty, digital rights, and data privacy protection have become the focus of research. Data are no longer traditional data. Data have become a factor of production in the field of production and an important part of digital economy; In the process of social transformation and development driven by digital economy, the digitization of productivity drives the overall digitization of production relations. In the field of daily life and consumption, data privacy, big data mining, and user imaging have become the focus of platform economy, ranging from individuals to enterprises and countries. Digital risks, digital ethics, and digital governance in the digital era have become the focus of academic circles, and interdisciplinary research by experts in multidisciplinary fields is urgently needed, providing constructive solutions for digital ethics and governance.

## Figures and Tables

**Table 1:** List of authors (number of articles ≥7) unit: article.

Sequence	Author	Volume of articles
1	Yudong Qi	25
2	Bing Chen	23
3	Dong Yang	20
4	Baoping Ren	16
5	Jiechang Xia	16
6	Yaojia Tang	13
7	Jinghua Xiao	12
8	Yuezhou Cai	11
9	Kang Xie	11
10	Zhanqi Yao	11
11	Fei Wu	10
12	Qichao Liu	9
13	Xianchun Xu	9
14	Daan He	9
15	Wenlong Han	9
16	Jian Dong Wang	8
17	XianMei Shi	8
18	Xiaohua Li	8
19	Chuxin Huang	8
20	Shuzhong Ma	8
21	Minfeng Lu	8
22	Cuihua Liu	8
23	Keyin Shen	8
24	Chang Cai	8
25	Yang He	7
26	Xiaheng Zhang	7
27	Jin Xu	7
28	Meihui Zhang	7
29	Min Wang	7
30	Mingxing Cao	7
31	Nian Li Zhou	7
32	Cheng Liu	7
33	Qingxin Lan	7
34	Yong jian Li	7
35	Qiaobin Feng	7
36	Bin Sheng	7
37	Dan Wang	6
38	Weiling Wang	6
39	Jin Chen	6
40	Dianchun Jiang	6
41	Xiaohong Chen	6
42	Lei Wang	6
43	Jin Sun	6
44	Shulei Ding	6
45	Changhong Pei	6
46	Guoquan Zang	6
47	Jianing Zheng	6
48	Xin Li	6
49	Yao Wu	6

**Table 2:** Statistical list of journal articles (number of articles ≥20) unit: articles.

Ordinal	Journal name	Volume of articles
1	*Journal of Commercial Economics*	100
2	*International Taxation in China*	98
3	*Taxation Research*	98
4	*People's Tribune*	74
5	*China Finance*	58
6	*Reform*	55
7	*Economist*	51
8	*E-Government*	50
9	*Southwest Finance*	46
10	*Intertrade*	42
11	*Price: Theory and Practice*	42
12	*Finance and Accounting Monthly*	40
13	*Statistics and Decision*	39
14	*Reform of Economic System*	38
15	*Economic Review Journal*	38
16	*China Business and Market*	31
17	*People's Tribune·Frontiers*	31
18	*Guizhou Social Sciences*	29
19	*Research on Financial and Economic Issues*	29
20	*Enterprise Economy*	26
21	*Science and Technology Management Research*	26
22	*Fiscal Science*	25
23	*Management World*	23
24	*Journal of Technical Economics and Management*	23
25	*Journal of Beijing Jiaotong University (Social Sciences Edition)*	23
26	*Statistical Research*	21
27	*Communication of Finance and Accounting*	20
28	*Asia-Pacific Economic Review*	20
29	*Macroeconomic Management*	20

**Table 3:** Statistical list of high-frequency keywords (threshold ≥20).

Ordinal	Keywords	Frequency
1	Digital economy	1660
2	High-quality development	233
3	Digital transformation	161
4	Data	100
5	Digital technique	100
6	Big data	87
7	Digital trade	86
8	Blockchain	77
9	Artificial intelligence	76
10	Digitization	67
11	Platform economy	58
12	Digital finance	56
13	Antitrust	55
14	COVID-19	54
15	Data governance	51
16	Industrial structure	51
17	Industrial digitization	49
18	Digital gap	49
19	Rural vitalization	44
20	Digital inclusive finance	43
21	Global value chain	42
22	Economic growth	41
23	Cross-border data flow	41
24	Data security	39
25	Financial technology	37
26	Digital platform	37
27	Digital governance	37
28	Digital service tax	35
29	Manufacturing	35
30	New development pattern	33
31	Tax administration	33
32	International tax rules	33
33	Digital currency	32
34	“The belt and road”	32
35	Technological innovation	31
36	Tax governance	31
37	Dual circulation	30
38	Digital industrialization	30
39	Internet	29
40	New infrastructure	29
41	Intermediary effect	28
42	Digital labor	27
43	Digital government	27
44	Internet platform	27
45	Digital village	26
46	Production factors	26
47	Innovation	26
48	International taxation	26
49	Electronic commerce	26
50	Total factor productivity	25
51	Common prosperity	25
52	Sharing economy	25
53	Digital age	24
54	European union	24
55	Network security	23
56	Real economy	22
57	Value creation	22
58	Global governance	22
59	Permanent establishment	22
60	Industrial chain	22
61	Personal information	21
62	New economy	21
63	Digital trade rules	21
64	Digital tax	21
65	Data assets	21
66	Value-added tax	20
67	Digital China	20

**Table 4:** High-frequency keyword co-occurrence matrix (part).

Keywords	Digital economy	High-quality development	Digital transformation	Data	Digital technique	Big data
Digital economy	1660	124	54	48	33	50
High-quality development	124	233	9	5	3	5
Digital transformation	54	9	161	6	8	7
Data	48	5	6	100	2	0
Digital technique	33	3	8	2	100	0
Big data	50	5	7	0	0	87

**Table 5:** High-frequency keyword dissimilarity matrix (part).

Keywords	Digital economy	High-quality development	Digital transformation	Data	Digital technique	Big data
Digital economy	−2.22045E−16	0.800616286	0.895545591	0.882188634	0.919004686	0.868430164
High-quality development	0.800616286	−2.22045E−16	0.953532254	0.967243911	0.980346347	0.964881786
Digital transformation	0.895545591	0.953532254	0	0.952713376	0.936951168	0.940853987
Data	0.882188634	0.967243911	0.952713376	0	0.98	1
Digital technique	0.919004686	0.980346347	0.936951168	0.98	0	1
Big data	0.868430164	0.964881786	0.940853987	1	1	1.11022E−16

## Data Availability

The experimental data used to support the findings of this study are available from the corresponding author upon request.
